# Combined Particulated Juvenile Articular Cartilage Allograft Transplantation With Autogenous Bone Graft for Symptomatic Osteochondral Defects in the Tibial Plateau

**DOI:** 10.1016/j.eats.2025.103689

**Published:** 2025-06-14

**Authors:** Paul B. Walker, Seth Cope, Rishi Trikha, Thomas J. Kremen, Kristofer J. Jones

**Affiliations:** Department of Orthopaedic Surgery, University of California, Los Angeles, Los Angeles, California, U.S.A.

## Abstract

Osteochondral lesions of the tibial plateau are rare, comprising only 1% to 2% of knee osteochondritis dissecans cases. Due to their rarity and challenging anatomic location, standardized treatment guidelines remain undefined. We describe a surgical technique combining autogenous bone grafting with particulated juvenile articular cartilage allograft (DeNovo NT; Zimmer Biomet). The advantages of this method include single-stage management, the presence of biologically active juvenile chondrocytes promoting robust hyaline-like cartilage, simultaneous restoration of subchondral bone defects, and enhanced surgical access to an anatomically challenging site.

Osteochondritis dissecans (OCD) is an acquired, idiopathic joint condition that affects the subchondral bone and articular surface.^1^^,^^2^ In the knee, most OCD lesions occur on the medial femoral condyle, followed by the lateral femoral condyle, patella, and trochlea.^3^ Lesions on the tibial plateau are exceedingly rare. Due to the infrequency of tibial plateau OCDs, there is a lack of established treatment protocols. Particulated juvenile articular cartilage (PJAC) allograft, marketed as DeNovo NT (Zimmer Biomet), presents a promising therapeutic option. Juvenile cartilage from donors aged 0 to 13 years is minced into 1-mm^3^ pieces, allowing nascent chondrocytes to infiltrate defects and form a hyaline-like matrix.^4^^,^^5^ Key advantages of this allograft include favorable gene expression, superior metabolic profiles compared to adult chondrocytes, availability as a fresh graft for single-stage procedures, ability to easily contour to the size and shape of the defect, and ability to deliver to difficult anatomic locations.^4^, ^5^, ^6^ This article presents a surgical technique utilizing autogenous bone graft and osteochondral allograft transplantation (DeNovo NT) as a potential treatment for these rare and complex lesions. Indications and contraindications for PJAC use are summarized in Table 1.^5^

**Table 1:** Indications and Contraindications Particulated Juvenile Allograft Cartilage Transplantation

Indications	Symptomatic articular cartilage defect in the patellofemoral or tibiofemoral compartment
	ICRS grade ≥3 lesions with minimal to no bone loss
	Patient age <55 years
	BMI <35
	Favorable physiologic age and remaining cartilage quality
**Absolute contraindications**	ICRS grade 1 or 2 lesions
	Meniscal deficiency
	Malalignment
	Uncorrected ligamentous instability
	OCD lesion with >6 mm subchondral bone loss (unless combined with bone grafting)
**Relative contraindications**	Bipolar lesions (risk of shearing)
	Bone marrow lesions (may be treated concurrently)
	Uncontained lesions or lesions with subchondral insufficiency (may require additional containment techniques or a sandwich bone grafting approach)
	

BMI, body mass index; ICRS, International Cartilage Repair Society; OCD, osteochondritis dissecans.

## Surgical Technique

**Fig 1:**
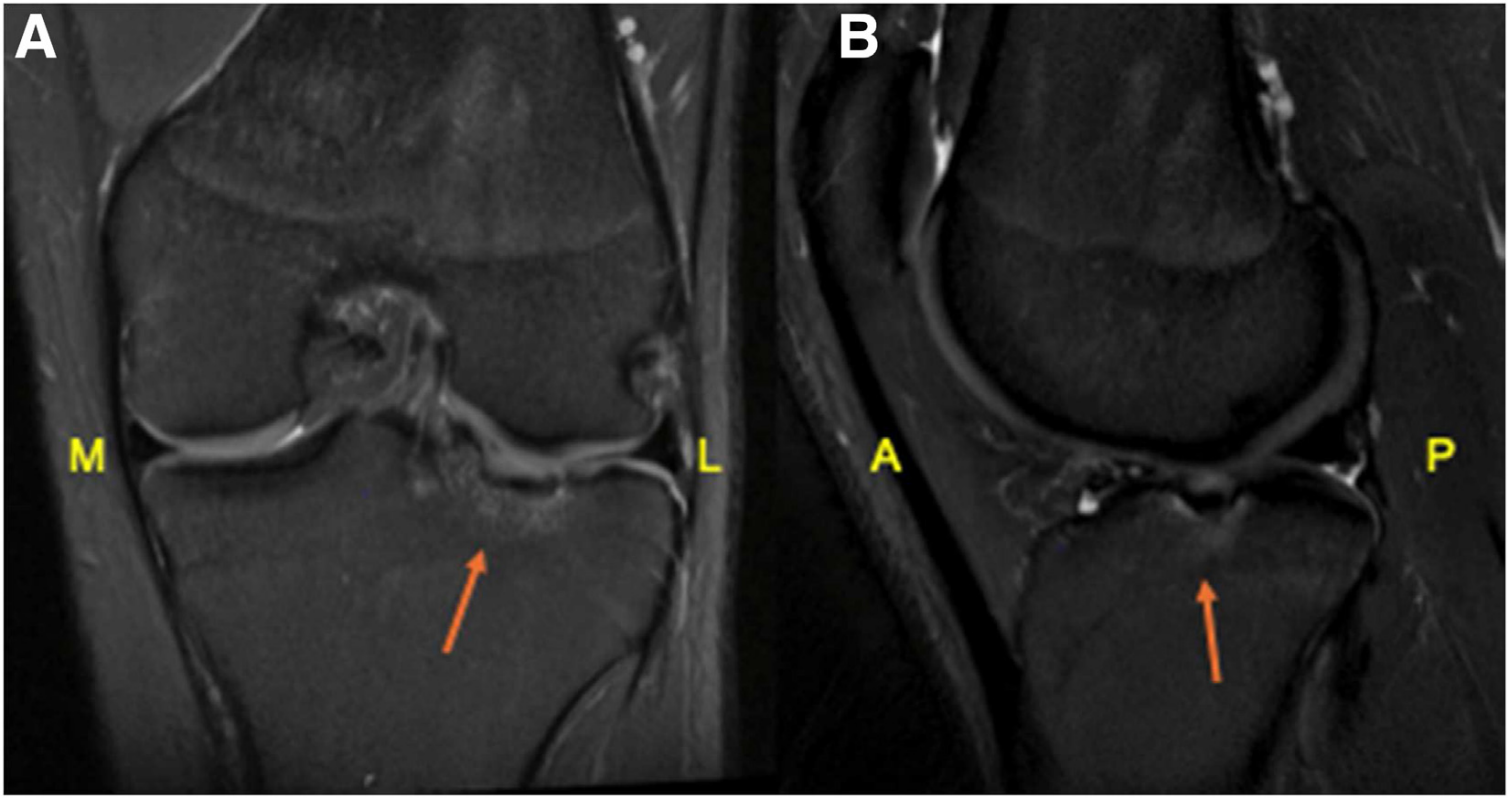
Preoperative magnetic resonance imaging showing an osteochondral defect measuring 16 × 16 × 5 mm in the central aspect of the lateral tibial plateau, shown on coronal (A) and sagittal (B) views. Red arrows indicate the defect location. Yellow labels denote anatomic orientation. (A, anterior; L, lateral; M, medial; P, posterior.)

Patients should undergo a comprehensive physical examination, followed by imaging studies. Standard radiographs, including alignment films, should be obtained to evaluate for fracture, advanced osteoarthritis, or malalignment. Magnetic resonance imaging (MRI) is essential to identify concomitant intra-articular pathologies that may require concurrent surgical management or represent contraindications to the procedure. Additionally, MRI provides detailed characterization of the osteochondral lesion, aiding in surgical planning (Fig 1).

In the operating room, the patient is positioned supine on the operating table with a lateral post. The leg is then prepped and draped in the usual sterile fashion, with a tourniquet applied high on the thigh.

**Fig 2:**
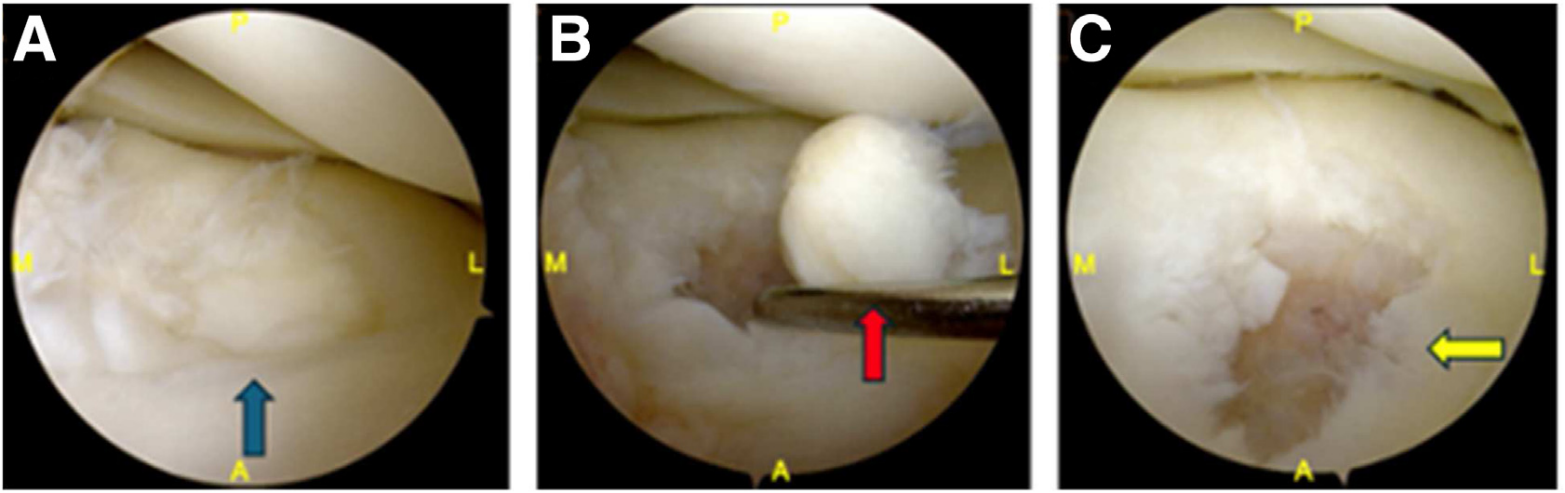
Diagnostic arthroscopy of the lateral tibial plateau. (A) An osteochondral defect is identified in the central portion of the lateral tibial plateau (blue arrow). (B) The unstable osteochondral fragment is probed and excised (red arrow); the lesion measured 16 × 16 mm. (C) A chondroplasty is performed using a No. 4-5 shaver, revealing the underlying sclerotic bone base (yellow arrow). (A, anterior; L, lateral; M, medial; P, posterior.)

**Fig 3:**
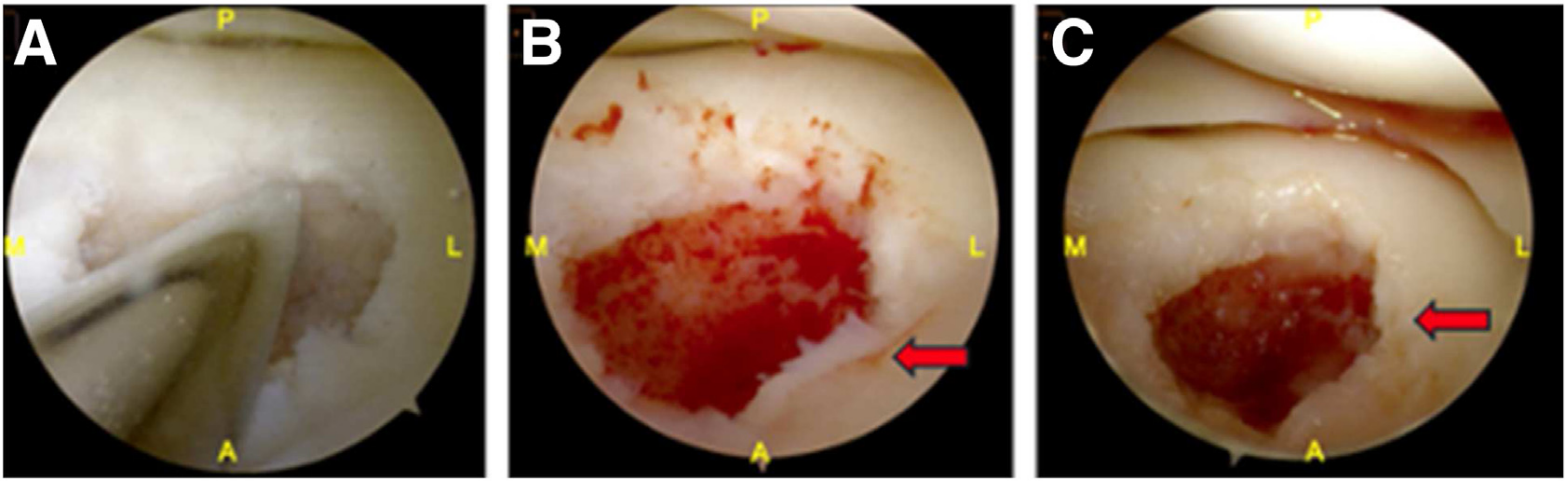
Arthroscopic preparation of the osteochondral defect. (A) The sclerotic lesion base is debrided using a handheld burr, revealing underlying bone loss. (B, C) The base is further prepared until punctate bleeding is observed, indicating a viable subchondral surface suitable for bone grafting. Red arrows highlight the bleeding bone bed. (A, anterior; L, lateral; M, medial; P, posterior.)

The procedure begins with a diagnostic arthroscopy performed using a standard 30° arthroscope introduced through anterolateral and anteromedial portals. This allows for a comprehensive assessment of concomitant intra-articular pathology and lesion characteristics that may contraindicate the use of DeNovo NT allograft, as outlined in Table 1. The osteochondral lesion is then probed to assess stability and excised using a grasper (Fig 2). Chondroplasty is performed with a No. 4-5 shaver to smooth the defect margins. The sclerotic base is meticulously debrided using a combination of a handheld burr and curette to achieve a stable and well-defined defect bed. Debridement should continue until punctate bleeding is observed from the subchondral bone, indicating a viable surface to support bone graft incorporation (Fig 3).

The proximal tibia is used to harvest bone grafts to address bone loss. A 3-cm incision is made over the anterior medial aspect of the proximal tibia to expose the tibial crest. An Osteoauger Bone Graft Harvesting System (Arthrex) is used to collect approximately 8 cc of autologous bone graft. This graft is mixed with 1 cc of demineralized bone matrix putty. To address the tibial void, cancellous allograft chips are inserted.

**Fig 4:**
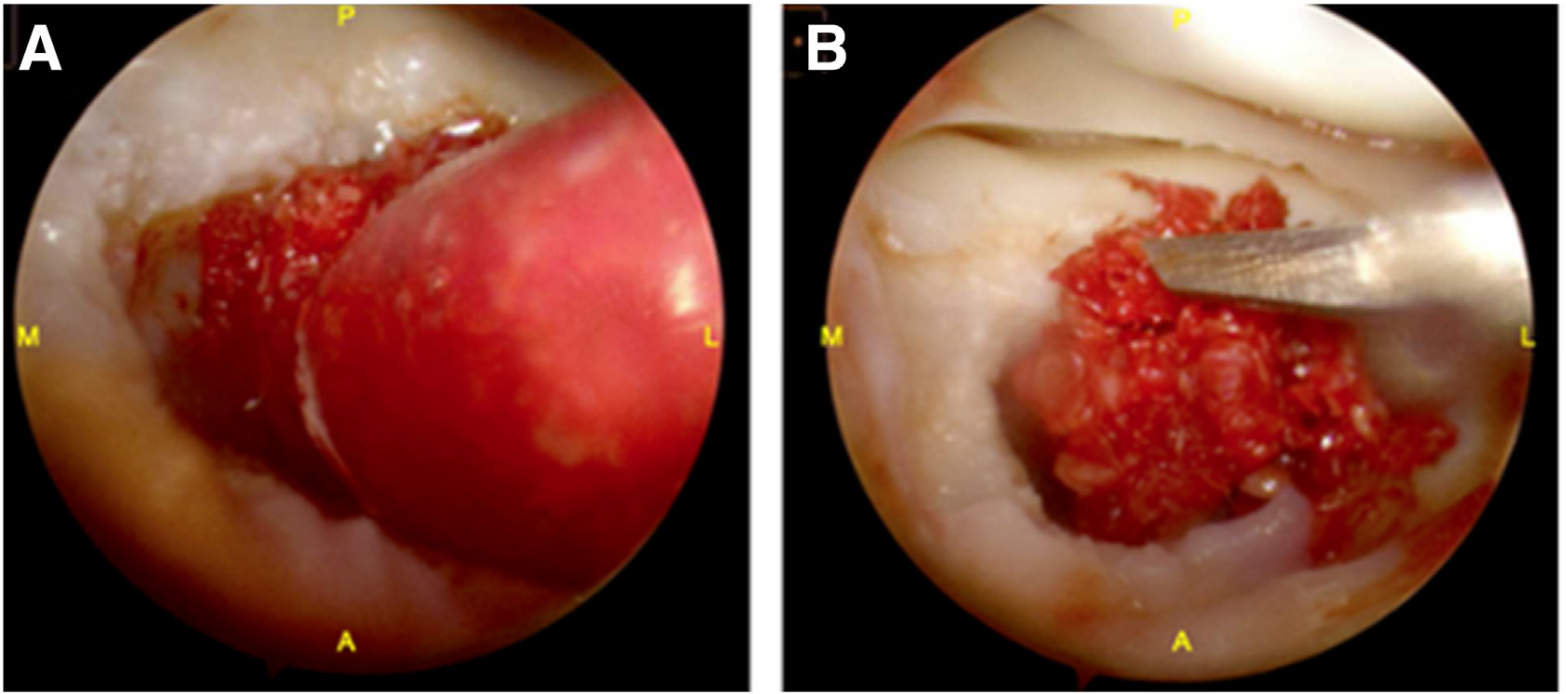
Bone grafting of the osteochondral defect. (A) Autologous bone graft is delivered to the defect using the BioXpress Graft Delivery Device (Arthrex). (B) The graft is manually packed with a large freer elevator and recessed to the level of the surrounding subchondral bone and articular surface. All instrumentation is introduced through a mini lateral arthrotomy, while visualization is maintained via an anteromedial arthroscopic portal. (A, anterior; L, lateral; M, medial; P, posterior.)

**Fig 5:**
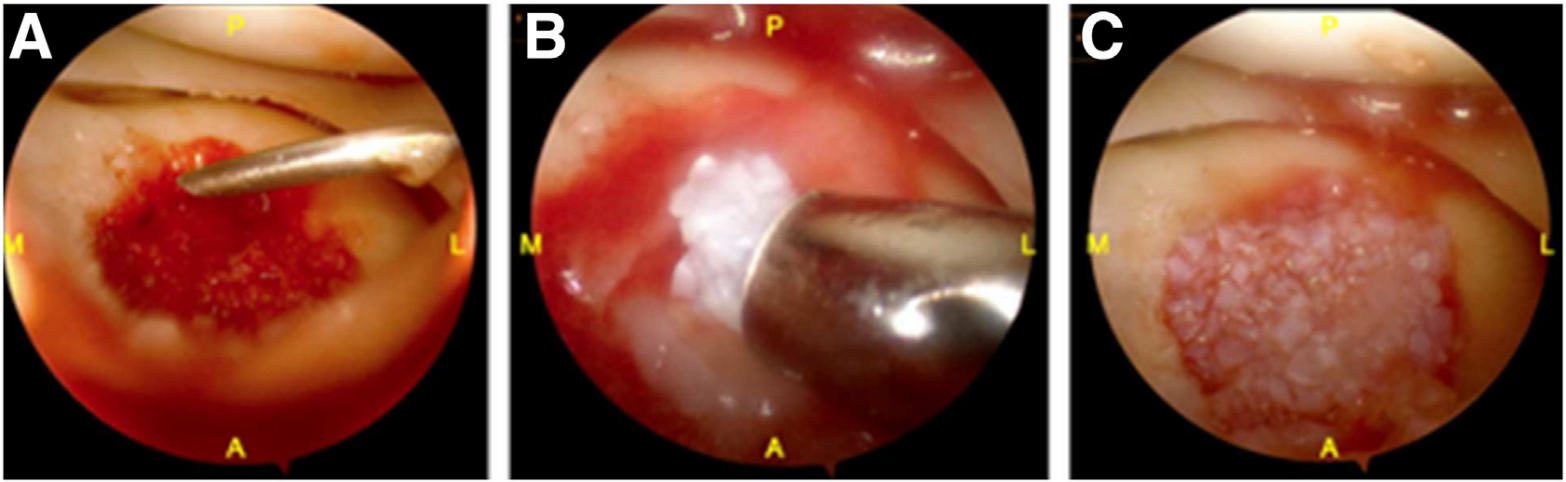
Delivery of particulated juvenile articular cartilage. (A) Fibrin glue is applied to the bone graft. (B) DeNovo NT (Zimmer Biomet) allograft is delivered using a Craig biopsy cannula. (C) A second layer of fibrin glue is applied to stabilize the construct. All instrumentation is introduced through a mini lateral arthrotomy, with visualization maintained through an anteromedial arthroscopic portal. (A, anterior; L, lateral; M, medial; P, posterior.)

Returning to the lateral tibial plateau, a 4-cm incision is made over the anterior knee, and a mini-lateral arthrotomy is performed. After partial excision of the infrapatellar fat pad, retractors are used to expose the lateral tibial plateau. The cartilage defect is clearly visualized using arthroscopy. The bone graft is meticulously delivered into the tibial plateau defect using the BioXpress Graft Delivery Device (Arthrex). It is then packed manually with a large freer elevator to ensure it is recessed to the level of the articular surface, extending up to the surrounding subchondral bone. Once the preparation is complete, fibrin glue is applied (Fig 4). We then use a Craig biopsy cannula to deliver the DeNovo NT allograft. Another layer of fibrin glue is applied, and a freer elevator is used to spread the mixture evenly across the defect to create a uniform monolayer scaffold (Fig 5). It is crucial to ensure the graft is level with the surrounding tissue to prevent graft hypertrophy and excessive shear forces that could jeopardize stability. Passive range of motion confirms secure graft placement. Pearls and pitfalls of the procedure are summarized in Table 2.

**Table 2:** Pearls and Pitfalls of PJAC and Bone Grafting for Tibial Plateau Osteochondritis Dissecans Lesions

**Pearls**
Preoperative imaging should include MRI and standing alignment radiographs to assess for malalignment and associated intra-articular pathology.
Perform diagnostic arthroscopy first to rule out contraindications.
Create a small lateral arthrotomy to facilitate passage of instrumentation and improve access to the tibial plateau.
Debride the lesion to a bleeding base using burr and curettes before bone grafting.
Harvest bone graft from the anterior proximal tibia using the OsteoAuger (Arthrex), which allows quick, high-volume autograft collection.
Mix autograft with demineralized bone matrix putty to improve handling and osteoconductivity during placement.
Use BioXpress Graft Delivery Device (Arthrex) to deliver the graft into deep or narrow defects.
Pack graft until flush with the surrounding subchondral bone using a freer elevator.
Deliver PJAC with a Craig biopsy cannula and gently spread into a monolayer with a freer elevator.
Use fibrin glue to stabilize the construct before and after PJAC application for mechanical protection and adherence.
Ensure graft is level with adjacent cartilage and perform passive range of motion to confirm mechanical stability prior to closing.
**Pitfalls**
Incomplete or irregular debridement can impair bone graft incorporation and chondrocyte adhesion.
Omitting fibrin glue risks graft displacement with irrigation or early motion.
Improper graft leveling or fill can cause joint incongruity, focal overload, graft hypertrophy, and eventual delamination or collapse.

MRI, magnetic resonance imaging; PJAC, particulated juvenile articular cartilage.

## Discussion

Tibial plateau OCD lesions are rare, accounting for approximately 1% to 2% of all knee OCD lesions.^7^ Most reported cases involve the lateral tibial plateau,^8^, ^9^, ^10^, ^11^, ^12^, ^13^, ^14^ while medial tibial plateau OCD lesions are less common.^15^ Due to the rarity of this condition and the challenging access to the tibial plateau, no standardized surgical approach exists. Techniques described in the literature include arthrotomy and excision, chondroplasty, microfracture, autologous chondrocyte implantation, calcium phosphate injection into the lesion, and retrograde osteochondral autograft procedure with meniscal centralization.^8^^,^^9^^,^^15^

While some providers may opt for excision alone, this approach increases the long-term risk of osteoarthritis (OA) and knee arthroplasty. A study by Sanders et al.^16^ highlighted that OCD fragment excision was associated with a significantly higher risk of OA and arthroplasty than fragment preservation or chondral defect grafting. Among 221 patients, the excision group had a 70% cumulative incidence of OA at 30 years, while no OA or need for arthroplasty was observed in the grafting group. These findings underscore the importance of a biologically restorative approach to prevent long-term joint degeneration.

We present a technique that combines autogenous bone grafting with PJAC for the treatment of OCD lesions of the tibial plateau. Although we are unaware of outcome studies reporting the use of PJAC for tibial plateau OCD, it has shown success in treating patellar OCD, where studies have reported favorable outcomes. In a study involving 36 knees with full-thickness patellar defects, treatment with PJAC resulted in a 100% return-to-sport rate, with 78% of grafts filling ≥50% of the defect depth, 64% achieving near-normal cartilage restoration, and 31% achieving complete defect fill on MRI.^17^ A similar investigation of 41 knees also reported a 100% return-to-sport rate, accompanied by improvements in short-term patient-reported outcomes.^18^ PJAC offers distinct advantages due to its use of live juvenile chondrocytes, which are highly metabolically active and capable of synthesizing substantial amounts of new cartilage matrix.^6^^,^^19^ This may lead to superior repair quality when compared to conventional allografts.^5^^,^^19^ Moreover, PJAC is sufficiently durable for use on load-bearing surfaces and can be applied to anatomically challenging areas, such as the tibial plateau.^5^

Specifically, in the context of tibial plateau OCD, the combination of autogenous bone grafting and PJAC offers distinct advantages over alternative surgical strategies. Microfracture alone often results in the formation of fibrocartilage and the creation of a large bone cavity, while bone grafting alone addresses bone loss but typically leads to fibrocartilage rather than hyaline-like cartilage.^20^, ^21^, ^22^, ^23^ This highlights the critical need to address both subchondral bone loss and cartilage defects concurrently. Compared to matrix autologous chondrocyte implantation, PJAC presents a more streamlined, single-stage approach, reducing surgical complexity and associated morbidity. In contrast, matrix autologous chondrocyte implantation may necessitate additional access and dissection, such as mobilizing the anterior horn of the lateral meniscus, to achieve sufficient exposure.^6^^,^^24^ Furthermore, conventional autograft and allograft plug techniques are fraught with technical challenges and higher surgical morbidity, particularly due to the limited access to the tibial plateau, where drilling plugs can be particularly difficult.^25^, ^26^, ^27^

While the use of PJAC combined with autologous bone graft offers a promising single-stage solution for tibial plateau OCD lesions, several limitations must be considered. The technique relies heavily on precise graft placement and stabilization; improper fill or leveling can result in joint surface incongruity, graft hypertrophy, or delamination. The absence of long-term outcome data for PJAC in tibial plateau applications raises uncertainty about durability, especially under high-load conditions. Additionally, this approach may not be suitable for patients with bipolar lesions, uncontained lesions, advanced cartilage degeneration, or significant malalignment, which could compromise integration and lead to early failure. Surgical access to the lateral tibial plateau remains technically challenging, and the procedure’s success is highly dependent on the surgeon’s familiarity with arthroscopic and mini-open techniques. Finally, the cost and availability of juvenile cartilage allograft may limit widespread adoption. Table 3 summarizes the advantages and disadvantages of autogenous bone grafting with PJAC.

**Table 3:** Advantages and Disadvantages of PJAC for Tibial Plateau Osteochondritis Dissecans Lesions

**Advantages**
A single-stage procedure that addresses both cartilage restoration and bone loss, potentially reducing overall surgical morbidity, recovery time, and operative costs
Juvenile chondrocytes are metabolically active and promote hyaline-like cartilage.
Bone graft and PJAC can be delivered to anatomically challenging or hard-to-access areas.
The procedure is performed using minimally invasive techniques.
The OsteoAuger (Arthrex) system efficiently morselizes a substantial volume of bone graft, facilitating immediate handling.
**Disadvantages**
Limited long-term outcome data for tibial plateau applications
Suboptimal for bipolar lesions, poor cartilage environments, or uncontained lesions
Cost of PJAC Potential pain over the proximal tibia graft harvest site

PJAC, particulated juvenile articular cartilage.

In summary, the combination of autogenous bone grafting and PJAC offers a promising approach with the potential for enhanced cartilage regeneration and improved functional recovery. PJAC is straightforward to implement, does not significantly increase surgical morbidity, and provides a higher likelihood of complete restoration, making it a valuable treatment option.

## Disclosures

The authors declare the following financial interests/personal relationships which may be considered as potential competing interests: T.J. Kremen serves as a board or committee member for the AAOS and the American Orthopaedic Society for Sports Medicine and is a member of the editorial or governing board for the *American Journal of Sports Medicine*. K.J. Jones is a paid consultant for Arthrex, JRF Ortho, and Vericel Corporation; receives research support from Aesculap/B. Braun, the Musculoskeletal Transplant Foundation, and Organogenesis; serves as a board or committee member for the American Orthopaedic Society for Sports Medicine, the *Journal of Bone and Joint Surgery* (*Am*), and the NFL Musculoskeletal Injury Committee; and holds stock or stock options in Sparta Biopharma. All other authors (P.W., S.C., R.T.) declare that they have no known competing financial interests or personal relationships that could have appeared to influence the work reported in this paper.

## Supplementary Data

Video 1 ▪▪▪
